# Effect of Social Networking Sites on the Quality of Life of College Students: A Cross-Sectional Study from a City in North India

**DOI:** 10.1155/2020/8576023

**Published:** 2020-05-01

**Authors:** Neeru Saini, Garima Sangwan, Madhur Verma, Adarsh Kohli, Manmeet Kaur, P. V. M. Lakshmi

**Affiliations:** ^1^Department of Community Medicine and School of Public Health, PGIMER, Chandigarh, India; ^2^Department of Community and Family Medicine, AIIMS Bathinda, Punjab, India; ^3^Department of Psychiatry, PGIMER, Chandigarh, India

## Abstract

**Introduction:**

With the advent and extensive use of the Internet and smartphones, social networking has become a pervasive part of human interaction. The use of these social networking sites or the Internet affects the physical, mental, and spiritual health of the people. Hence, there is need to understand how the time spent on social networking is affecting the quality of life (QOL) as a whole, especially among college-going students who are most likely users of social networking sites (18–21 years).

**Materials and Methods:**

A cross-sectional survey was conducted among 220 college-going students (18–21 years) in Chandigarh in 2012. The data were collected using a pretested self-administered questionnaire, adapted from Young's Internet usage questionnaire. Appropriate statistical analysis was done.

**Results:**

Almost all (98%) of the respondents use the Internet. As compared to nondaily users of social networking sites, daily users were better able to handle stress related to (1) relationships (moderate to severe stress among daily users vs. nondaily users, 15.2% vs. 30.5%) and (2) work (moderate to severe stress among daily users vs. nondaily users, 18.2% vs. 35.4%). The daily users of social networking sites feel significantly more satisfied with their classmates, the way they handle the problems, their physical appearance, and their accomplishments in their life.

**Conclusion:**

Social networking sites are steadily penetrating in the lives of adolescents in India. The advantages on quality of life for daily users of social networking sites versus nondaily users are enormous. Also currently, Internet use might not have reached the levels where it embarks on the existing state of health; therefore, continuous and critical observation of the changing trends is warranted.

## 1. Introduction

Social network sites are web-based services that allow individuals to construct a public or semipublic profile within a bounded system, articulate a list of other users with whom they share a connection, and view and traverse their list of connections and those made by others within the system [1]. Adolescents are one of the major age groups for the use of social networking sites [2].

With the advent and extensive use of the Internet and smartphones, social networking has become a pervasive part of human interaction and is changing the societal rules in the modern world at a tremendous pace. At least 8 billion minutes are spent on Facebook each day. One of the reasons Facebook is so addictive is because it is a convenient way to track the life of your friends. India has the second largest online market in the world. The number of Internet users in India has increased from 259.88 million in 2015 to 437.4 million in 2017. This figure is projected to grow to 525.3 million Internet users in 2019 [3].

The use of these social networking sites or Internet affects the physical, mental, and spiritual health of the people. Social networking has provided an indispensable method of communication and ignores geographical and cultural barriers. On a positive note, it improves health and professional life by giving information on health, improving eye-hand coordination, providing information on career options, timely work-related data transmission [4].

Besides this, it has some adverse effects also like cyberbullying and crimes against children, risks of fraud or identity theft, and invasion in privacy. Adolescents spending more and more time on the Internet and social networking sites (SNSs) are prone to restrict physical activities, obesity, insomnia, eyesight-related problems, body aches, and so forth. It is seen the adolescents with use of Internet are at more risk of sexting and risky sexual behaviours and are more prone to drug addiction [5]. Though SNSs allow people to create new relationships and reconnect with friends and family, time spent on face to face socialization is being jeopardized. Parents spend less time with their children even when they are living in the same home [6].

Tiredness due to long school hours, commuting times, and threats of crime act as a compelling factor for holding adolescents back at home for long hours that restricts physical and social interaction among them. Due to this active user had shown a tendency to develop social isolation, which ultimately affects the interpersonal relationships [7].

The recent reports of blue whale deaths from various parts of India especially among the adolescents and young adults were also related to using SNSs.

Hence, there is a need to understand how the time spent on social networking is affecting the quality of life (QOL) as a whole, especially among college-going students who are most likely users of SNSs (18–21 years). QOL is a multipronged concept that points towards an individual's overall well-being and life satisfaction in the context of culture and value systems and about goals, expectations, standard, and concerns [8].

College-going students are more curious and vulnerable to be affected by the physical, mental, social, and psychological changes happening in and around them as they get more freedom from parental and familial influences once they leave school and enter a whole new phase of life. Most of the studies on social networking and its impact on the QOL have been conducted in the West, where the Internet revolution has occurred earlier, and the social milieu of those countries is different from that of India. Thus, there it is crucial to study the association of usage of SNSs with the QOL.

## 2. Methodology

A cross-sectional survey was conducted among college-going students (18–21 years) in Chandigarh in 2012. After a review of the literature, a sample size of 220 was calculated at power of 80% and 95% confidence assuming that 20% of daily users will have some problems as compared to 10% among nonregular users. The sample size attained was 63 in each group. The sample size was increased to 220 so that we have at least 63 nonregular users in our study.

The list of government, private, and government aided colleges was obtained from Director of Higher Education, colleges (DHE), and all colleges (*N* = 14) were stratified into boy's colleges (*N* = 3), girls colleges (*N* = 5), and coeducation colleges (*N* = 6). From each of the strata, 1–2 colleges were selected randomly (girls college, 1, boys college, 1, and coed. Colleges, 2). Prior permission was obtained from DHE colleges and the principals of the selected colleges for conducting the survey. Two classes were randomly allotted by the college administration from each of the four colleges. All students in the selected classes were invited for the survey, out of which the first 55 eligible respondents from each class were included in the study. Students older than 21 years of age were excluded.

After explaining the purpose of the study, written informed consent was taken. The confidentiality of the data was assured, and the data was collected using a pretested self-administered questionnaire. The questionnaire has three sections.  Section 1 has 20 items collecting information on sociodemographic details of the subjects.  Section 2 had 17 items collecting information on the status of the Internet and SNS usage, length of usage, and the frequency of various activities done on the Internet. The questions in this section were adapted from Young's Internet usage questionnaire [9]. Also, questions regarding usage of SNSs, number and name of the most frequently visited sites, and the three best and worst things about the Internet were also included.  Section 3 had six subsections related to QOL, namely, physical state (6 items), mental state (10 items), stress evaluation (10 items), life enjoyment (10 items), the perceived overall quality of life (11 items), and overall impression about all the five domains (5 items) [10].

Ethical clearance was obtained from the Institutional ethical review committee. Pretesting was done among 20 students of the same age group who were not included in the study. The data was collected using a supervised, pretested, semistructured, and self-administered questionnaire in a classroom after giving standardized instructions for filling the form to the students.

For the study, the social networking site users were classified as daily and nondaily users. Daily users were defined as those who were using social networking sites every day, and nondaily users were using social networking sites but not daily.

Average time spent on social networking sites, perceived quality of life (QOL), and baseline sociodemographic information were presented as means and percentages. The association between perceived QOL and the use of social networking sites was tested using *χ*^2^ test.

## 3. Results

The mean age of the study participants (*N* = 220) was 19.3 years. Among the respondents 56% were females. Most of the respondents (80%) belonged to a nuclear family. Only about a quarter (24%) of the families had both parents working. About three-fifths of the respondent's study arts and humanities (62%) and most of them (94%) do not have any other source of income except their pocket money. Only 5% of the respondents said they do not have friends.

Almost all (98%) of the respondents use the Internet. The most common reason for using the Internet was social networking (57%) followed by chatting (22%) and listening to music or sharing files (17%). The best thing the respondents like about the Internet was connecting with the friends (67%) followed by getting information and downloading (48%). Wastage of time (31%) and hacking (20%) were the most common side effects of the Internet by respondents.

Among Internet users, 63% were daily users of SNS. The association of sociodemographic variables with daily and nondaily users of the Internet is presented in Table 1. There was no significant association of daily use of the Internet with sociodemographic characteristics except for pocket money (*p* value: 0.003). The proportion of daily users increased with the increase in the amount of pocket money the respondents got every month (*χ*^2^ test for trend: 10.11; *p* value: 0.0015).

Facebook was found to be the most popular SNS (97%) that respondents were using. When respondents were asked about their way of communicating with friends other than through social networking sites, 80% of the respondents preferred to communicate using WhatsApp. More than half of the respondents had online contacts whom they had never met in the real world. About half of the respondents (55%) had met someone in real after contacting them online, and one-third of them were accompanied by their friends or parents during such meetings.

SNSs were rarely (3%) accessed during school hours. More than half of the respondents (56%) use mobile phones for using the Internet and social networking sites and about one-third (35%) access SNS at home.

**Figure 1:**
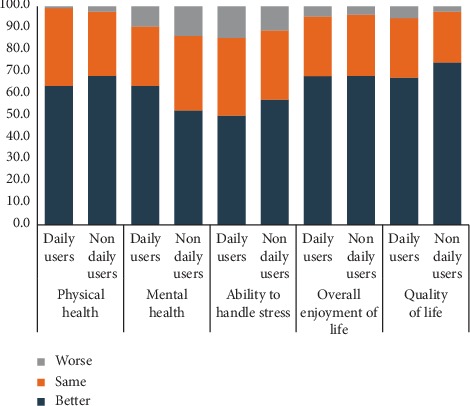
Overall impression about health among social networking site users in Chandigarh.

There were no significant differences in the mental and physical health problems reported by the daily and nondaily users of SNSs (Table 2). Though there were no significant differences in the overall impression of health perceived by daily users and nondaily users (Figure 1), there were significant differences in few of the domains related to the stress perceived (Table 3), life satisfaction (Table 4), and the overall quality of life perceived (Table 5). As compared to nondaily users of social networking sites, daily users were better able to handle stress related to relationships (moderate to severe stress among daily users vs. nondaily users: 15.2% vs. 30.5%; (*p* value: 0.03) and work (moderate to severe stress among daily users vs. nondaily users: 18.2% vs. 35.4%; *p* value –0.01). In the rest of the domains related to perceived stress, there were no significant differences. The level of life satisfaction in the domains of feeling of joy (mo or slight feeling of joy among daily users vs. nondaily users: 17% vs. 26%; *p* value: 0.04) and time devoted to things one enjoys (no or less time among daily users vs. nondaily users: 21% vs. 38%; *p* value: 0.003) were higher among daily users as compared to nondaily users. In the rest of the domains of life satisfaction, there were no significant differences among daily users and nondaily users of social networking sites. The daily users of social networking sites feel significantly more satisfied with their classmates (daily users vs. nondaily users: 67% vs. 55%; *p* value: 0.010), the way they handle the problems (daily users vs. nondaily users: 53% vs. 32%; *p* value: 0.003), their physical appearance (daily users vs. nondaily users: 62% vs. 45%; *p* value: 0.02), and their accomplishments in their life (daily users vs. nondaily users: 55% vs. 37%; *p* value: 0.02).

## 4. Discussion

The present study focused primarily on the prevalence of use of SNSs and its effect on the quality of life among college-going adolescents of Chandigarh, India. The maximum number of respondents (98%) had access to the Internet and SNSs. It is an essential part of their daily activities as 58% of respondents reported that they were logging onto social networking sites at least once a day. Basic sociodemographic variables including gender, age, and family type were comparable among daily and nondaily users of the SNSs. Therefore, these variables are unlikely to confound the relationship between social networking and the perceived quality of life and therefore were not adjusted using multivariable analysis. The pattern of use is similar to students of western countries, and it was observed that around 87% of teenagers from these countries log onto their favourite SNS, while 55–64% of adolescents have an online profile and more than half log more than once a day [2].

Mobile phones (56%) were observed to be the most common medium for accessing the Internet and SNSs, but students avoided logging into their accounts during the schools hours or in computer classes (3%). This is a good habit observed that is similar to many other studies where the home was chosen as the most preferred place for accessing the Internet by adolescents worldwide [11]. A positive correlation was observed between the pocket money and the extent of daily usage of SNSs. This is justified by the fact that the majority of them accessed SNSs through smartphones (56%) which require money for paying the carrier service charges. Students with restricted pocket money tend to use mobile data cautiously, and SNSs are less frequently accessed.

There were no significant differences observed in the prevalence of physical and social health problems among daily and nondaily users of the SNSs. This is in contrast to studies done among adolescents from developed countries, who were regularly accessing the Internet and SNSs from last 5 to 6 years and have reported posture related problems and headache [12]. This could be the future state of our respondents as currently, Internet use might not have reached the levels where it embarks on the existing state of health. A cross-sectional study in a rural area in Japan aimed to examine the relationship between problematic Internet use (PIU) and psychological distress, insomnia, and alcoholism among schoolteachers. The results indicate that schoolteachers with more severe psychological distress, insomnia, or alcoholism, regardless of gender, would use the Internet in a more problematic way [13]. Study on the quality of life (QoL) of older adults in Portugal reveals the importance of the Internet on the QoL of older adults, in particular, the fact that this technology optimizes the positive impact of confidant networks on QoL [12].

As compared to nondaily users of social networking sites, daily users were better able to handle the stress related to relationships and work. This is supported by the hypothesis that some people prefer to communicate online as compared to face to face communication due to greater anonymity and less social perceived risk [14]. Social support has been seen as the most effective buffer to stress, and SNSs present a potential intervention opportunity for developing and strengthening supportive social networks for vulnerable individuals. SNSs may help the users to vent out their feeling in front of their peers and feel better. This explains more stress among nondaily users that is especially related to work and relationships as compared to daily users.

In contrast, an online survey of adults 18–49 of age, to assess the independent associations between SNS use and depressive symptoms using ordered logistic regression, found each one-point increase in SNS use was associated with a 33 percent increase in depressive symptoms (adjusted odds ratio (AOR) = 1.33, 95 percent confidence interval (CI) = 1.17–1.51) [15]. Also, a longitudinal panel study among 12–19-year-old Flemish adolescents to investigate the reciprocal relationships between different types of Instagram use and depressed mood reported data from 671 adolescent Instagram users and showed that Instagram *browsing* at time 1 was related to increases in adolescents' depressed mood at time 2 and vice versa [16]. In the present study, nondaily users of SNSs were not at all satisfied by the less frequent experience of joy and time devoted to the things they enjoyed. It might be because of the restricted access to the Internet and SNSs resulting in poor connectivity with the peers for sharing their problems and poor social capital among them. Socially isolated people tend to experience more stress and are more prone to accidents; therefore, they are at greater risk of developing psychiatric disorders, hypertension, heart disease, and so forth, leasing to shorter life span [14].

The importance of online social networking in building social capital has been highlighted in our study whereas previous literature considered offline or face to face social networking superior. This can be attributed to an increase in like-minded people in our society who do not mind being approached through online networks [17]. This is also supported by the fact that around 68% of daily users in our study were satisfied by their relationships with their classmates as compared to nondaily users. More than half (55%) of daily users were satisfied by their way of handling the problems. This can be attributed to a healthy discussion of problems in the peer groups that help in reaching better solutions. Also, there is an improvement in the experience of handling problems of their friends that helps them too. Among international students in China, loneliness was found to be the strongest predictor for smartphone addiction [18]. Daily users perceived that they were more satisfied with their accomplishment in life (55%) and their physical appearance (62%) as compared to nondaily users. The reason might be that the virtual world is enabling them to protect themselves in their own ways and hence they can overcome their shortcomings and their inhibitions [17]. But this may be a vicious cycle as SNS use may be reinforced by experienced gratification and relief from negative feelings, and hence it may lead to addiction with long term use. In a study among a total of 511 SNS using young adults aged 20–35 years recruited online, about 4.9% (*n* = 25) of all participants could be classed as having a high SNS addiction risk profile.

Moreover, the results further indicated that fear of missing out (*β* = 0.38), maladaptive cognitions (*β* = 0.25), and psychiatric distress (*β* = 0.12) significantly predicted SNS addiction (i.e., *p* < 0.0001) [19]. The QOL observed among the study participants was independent of the time spent on the Internet including the SNSs. These results were consistent with observations from the past [20]. SNSs are steadily penetrating in the lives of adolescents in India. SNSs increase social capital and help them communicate with peers [4]. The advantages are enormous, but the users are still in their initial stages as compared to the western world, it is speculated that overuse might shift them from positive social capital to addiction and related problems. It is proven that less intimate and short-term relationships through Internet communication cause loneliness and ignoring other stress reducing and friends making activities in the real world add to the existing problem [7]. Continuous and critical observation of the changing trends is therefore warranted.

## 5. Strengths of the Study

Most of the earlier studies were conducted in developed countries with different sociocultural background from that of India. This study is, therefore, one of its kind from India which explored the effect of frequency of social networking on the quality of life of college-going adolescents.

## 6. Conclusion

The study suggests various advantages on quality of life for daily users of social networking sites versus nondaily users. Daily users are better able to handle stress related to relationships and work; they are more satisfied with their classmates, the way they handle the problems, and their physical appearance. The prevalence of physical and social health problems among daily and nondaily users of the SNSs showed no significant difference. Currently, Internet use might not have reached the levels where it embarks on the existing state of health. Continuous and critical observation of the changing trends is therefore warranted.

## Figures and Tables

**Table 1:** Demographic characteristics of social networking sites users among college-going adolescents of Chandigarh.

Characteristics		Total users	Daily users	Nondaily users	*p* value
Gender	Male	96	63 (65.6)	33 (34.4)	0.2
	Female	118	69 (58.65)	49 (41.5)	

Family structure	Joint	42	28 (66.7)	14 (33.3)	0.46
	Nuclear	172	104 (60.5)	68 (39.5)	

Education	Engineering and science	84	51 (60.7)	33 (39.3)	0.82
	Arts and humanities	130	81 (62.3)	49 (37.7)	

Religion	Hindu	173	106 (61.3)	67 (38.7)	0.90
	Sikh	34	22 (64.7)	12 (35.5)	
	Others	7	4 (57.1)	3 (42.9)	

Pocket money	<500	27	10 (37)	17 (63)	0.003
	501–2500	90	50 (55.6)	36 (41.9)	
	>2501	103	72 (69.9)	29 (28.7)	

Other sources of income	Yes	13	8 (61.5)	5 (38.5)	1
	No	201	124 (61.7)	77 (38.3)	

Who they live with	Alone	62	43 (69.4)	19 (30.6)	0.14
	With parents	152	89 (58.6)	63 (41.4)	

No. of family members	1–5	186	114 (61.3)	72 (38.7)	0.76
	>5	28	18 (64.3)	11 (35.7)	

Do they have siblings	Yes	173	102 (59)	71 (41)	0.09
	No	41	30 (73)	11 (26.8)	

Peer group (friends)	Yes	203	127 (62.6)	76 (37.4)	0.26
	No	11	5 (45.5)	6 (54.5)	

**Table 2:** Association of physical and mental health problems with use of social networking sites among adolescents in Chandigarh, India.

Problems		Daily users (%)	Nondaily users (%)	*p* value
Physical health				
Presence of physical pain	Never/rarely	88 (66.7)	63 (76.8)	0.22
	Occasionally	40 (30.3)	16 (19.5)	
	Regularly/constantly	4 (3.0)	3 (3.7)	

Feeling of tension	Never/rarely	75 (56.8)	37 (47.6)	0.40
	Occasionally	44 (33.3)	32 (39)	
	Regularly/constantly	13 (9.8)	11 (13.4)	

Lack of physical energy	Never/rarely	87 (65.9)	51 (62.2)	0.84
	Occasionally	30 (22.7)	20 (24.4)	
	Regularly/constantly	15 (11.4)	11 (13.4)	

Incidence of headache	Never/rarely	82 (62.1)	58 (70.7)	0.32
	Occasionally	36 (27.3)	15 (18.3)	
	Regularly/constantly	14 (10.6)	9 (11)	

Incidence of dizziness	Never/rarely	106 (80.3)	68 (82.9)	0.31
	Occasionally	19 (14.4)	13 (15.9)	
	Regularly/constantly	7 (5.3)	1 (1.2)	

Incidence of accidents	Never/rarely	115 (87.1)	78 (95.1)	0.08
	Occasionally	11 (8.3)	4 (4.9)	
	Regularly/constantly	6 (4.5)	0 (0)	

Mental health status				
Distress about pain	Never/rarely	88 (66.7)	61 (74.4)	0.46
	Occasionally	25 (18.9)	13 (15.9)	
	Regularly/constantly	19 (14.4)	8 (9.8)	

Presence of negative feelings	Never/rarely	89 (67.4)	48 (58.5)	0.42
	Occasionally	31 (23.5)	24 (29.3)	
	Regularly/constantly	12 (9.1)	10 (12.2)	

Experience of moodiness	Never/rarely	56 (42.4)	35 (42.7)	0.92
	Occasionally	49 (37.1)	32 (39.0)	
	Regularly/constantly	27 (20.5)	15 (18.3)	

Experience of low feeling	Never/rarely	67 (50.8)	48 (58.5)	0.50
	Occasionally	41 (31.1)	20 (24.4)	
	Regularly/constantly	24 (18.2)	14 (12.1)	

Overly worried about small things	Never/rarely	74 (56.1)	47 (57.3)	0.89
	Occasionally	27 (20.5)	18 (22)	
	Regularly/constantly	31 (23.5)	17 (20.7)	

Difficulty in concentration	Never/rarely	79 (59.8)	38 (47.5)	0.16
	Occasionally	33 (25)	23 (28.8)	
	Regularly/constantly	20 (15.2)	19 (23.8)	

Vague fears	Never/rarely	99 (75.0)	60 (74.1)	1
	Occasionally	22 (16.7)	14 (17.3)	
	Regularly/constantly	11 (8.3)	7 (8.6)	

Experience of restlessness	Never/rarely	89 (67.4)	55 (67.1)	0.99
	Occasionally	26 (19.7)	16 (19.5)	
	Regularly/constantly	17 (12.9)	11 (13.4)	

Difficulty in sleeping	Never/rarely	100 (75.8)	61 (74.4)	0.92
	Occasionally	23 (17.4)	16 (19.5)	
	Regularly/constantly	9 (6.8)	5 (6.1)	

Experience of recurring thoughts	Never/rarely	80 (60.6)	48 (58.5)	0.75
	Occasionally	33 (25)	19 (23.2)	
	Regularly/constantly	19 (14.4)	15 (18.3)	

**Table 3:** Association of perceived stress with use of social networking sites among adolescents in Chandigarh, India.

Problem		Daily users (%)	Nondaily users (%)	*p* value
Family	Never/slight	99 (75)	57 (70.4)	0.42
	Moderate	25 (18.9)	15 (18.5)	
	Pronounced/extensive	8 (6.1)	9 (11.1)	

Significant relationship	Never/slight	90 (68.2)	46 (56.1)	0.03
	Moderate	20 (15.2)	25 (30.5)	
	Pronounced/extensive	22 (16.7)	11 (13.4)	

Health	Never/slight	86 (65.2)	54 (65.9)	0.12
	Moderate	31 (23.5)	12 (14.6)	
	Pronounced/extensive	15 (11.4)	16 (19.5)	

Finances	Never/slight	78 (59.1)	49 (59.8)	0.73
	Moderate	28 (21.2)	20 (24.4)	
	Pronounced/extensive	26 (19.7)	13 (15.9)	

Work	Never/slight	83 (62.9)	39 (47.6)	0.02
	Moderate	24 (18.2)	29 (35.4)	
	Pronounced/extensive	25 (18.9)	14 (17.1)	

College	Never/slight	72 (54.5)	36 (43.9)	0.32
	Moderate	31 (23.5)	24 (29.3)	
	Pronounced/extensive	29 (22)	22 (26.8)	

Friends	Never/slight	96 (72.7)	51 (62.2)	0.09
	Moderate	17 (12.9)	20 (24.4)	
	Pronounced/extensive	19 (14.4)	11 (13.4)	

General well-being	Never/slight	88 (66.7)	61 (74.4)	0.23
	Moderate	34 (25.8)	13 (15.9)	
	Pronounced/extensive	10 (7.6)	8 (9.8)	

Emotional well-being	Never/slight	75 (56.8)	42 (51.2)	0.63
	Moderate	40 (30.3)	26 (31.7)	
	Pronounced/extensive	17 (12.9)	14 (17.1)	

Copying with daily activities	Never/slight	87 (66.4)	53 (64.6)	0.85
	Moderate	36 (27.5)	25 (30.5)	
	Pronounced/extensive	8 (6.1)	4 (4.9)	

**Table 4:** Association of life satisfaction with use of social networking sites among adolescents in Chandigarh, India.

Life satisfaction dimension		Daily users (%)	Nondaily users (%)	*p* value
Openness to guidance to inner voice	Not at all/slight	48 (36.4)	35 (42.7)	0.60
	Moderate	73 (55.3)	42 (51.2)	
	Considerable/extensive	11 (8.3)	5 (6.1)	

Feeling of well-being	Not at all/slight	33 (25)	28 (34.1)	0.22
	Moderate	80 (60.6)	47 (57.3)	
	Considerable/extensive	19 (14.4)	7 (8.5)	

Presence of positive feelings	Not at all/slight	32 (24.2)	27 (32.9)	0.07
	Moderate	70 (53)	46 (56.1)	
	Considerable/extensive	30 (22.7)	9 (11)	

Interest in maintaining healthy life style	Not at all/slight	24 (18.2)	16 (19.5)	0.82
	Moderate	74 (56.1)	48 (58.5)	
	Considerable/extensive	34 (25.8)	18 (22)	

Feeling of awareness to surrounding	Not at all/slight	32 (24.2)	21 (25.6)	0.39
	Moderate	77 (58.3)	41 (50)	
	Considerable/extensive	23 (17.4)	20 (24.4)	

Level of confidence in dealing with adverse condition	Not at all/slight	28 (21.2)	18 (22)	0.14
	Moderate	82 (62.1)	58 (70.7)	
	Considerable/extensive	22 (16.7)	6 (7.3)	

Level of compassion	Not at all/slight	32 (24.2)	21 (25.6)	0.96
	Moderate	80 (60.6)	48 (58.5)	
	Considerable/extensive	20 (15.2)	13 (15.9)	

Satisfaction with the level of recreation	Not at all/slight	36 (27.3)	24 (29.3)	0.06
	Moderate	78 (59.1)	55 (67.1)	
	Considerable/extensive	18 (13.6)	3 (3.7)	

Incidence of feeling of joy	Not at all/slight	23 (17.4)	21 (25.6)	0.04
	Moderate	82 (62.1)	54 (65.9)	
	Considerable/extensive	27 (20.5)	7 (8.5)	

Time devoted to things enjoyed	Not at all/slight	28 (21.5)	31 (38.3)	0.003
	Moderate	76 (58.5)	45 (55.6)	
	Considerable/extensive	26 (20)	5 (6.2)	

**Table 5:** Association of overall quality of life with use of social networking sites among adolescents in Chandigarh, India.

QOL dimension		Daily users (%)	Nondaily users (%)	*p* value
Personal life	Terrible/unhappy/dissatisfied	17 (12.9)	12 (14.6)	0.37
	Mixed	41 (31.1)	32 (39)	
	Mostly satisfied/pleased/delighted	74 (56.1)	38 (46.3)	

Romantic life	Terrible/unhappy/dissatisfied	43 (32.6)	35 (42.7)	0.08
	Mixed	32 (24.2)	24 (29.3)	
	Mostly satisfied/pleased/delighted	57 (43.2)	23 (28)	

College	Terrible/unhappy/dissatisfied	19 (14.4)	13 (15.9)	0.27
	Mixed	39 (29.5)	32 (39)	
	Mostly satisfied/pleased/delighted	74 (56.1)	37 (45)	

Classmates	Terrible/unhappy/dissatisfied	21 (15.9)	8 (9.8)	0.01
	Mixed	23 (17.4)	29 (35.4)	
	Mostly satisfied/pleased/delighted	88 (66.7)	45 (54.9)	

Actual work done	Terrible/unhappy/dissatisfied	18 (13.6)	17 (20.7)	0.25
	Mixed	39 (29.5)	27 (32.9)	
	Mostly satisfied/pleased/delighted	75 (56.8)	38 (46.3)	

Handling of problems	Terrible/unhappy/dissatisfied	22 (16.7)	28 (34.1)	0.003
	Mixed	40 (30.3)	28 (34.1)	
	Mostly satisfied/pleased/delighted	70 (53)	26 (31.7)	

Accomplishment in life	Terrible/unhappy/dissatisfied	21 (15.9)	22 (26.8)	0.02
	Mixed	38 (28.8)	30 (36.6)	
	Mostly satisfied/pleased/delighted	73 (55.3)	30 (36.6)	

Physical appearance	Terrible/unhappy/dissatisfied	14 (10.6)	18 (22)	0.02
	Mixed	36 (27.3)	27 (32.9)	
	Mostly satisfied/pleased/delighted	82 (62.1)	37 (45.1)	

Adjustment with changes in life	Terrible/unhappy/dissatisfied	18 (13.6)	19 (23.2)	0.17
	Mixed	40 (30.3)	25 (30.5)	
	Mostly satisfied/pleased/delighted	74 (56.1)	38 (46.3)	

Satisfaction with life	Terrible/unhappy/dissatisfied	26 (19.7)	15 (18.3)	0.63
	Mixed	40 (30.3)	30 (36.6)	
	Mostly satisfied/pleased/delighted	66 (50)	37 (45.1)	

Life as a whole	Terrible/unhappy/dissatisfied	20 (15.2)	12 (14.6)	0.34
	Mixed	33 (25)	28 (34.1)	
	Mostly satisfied/pleased/delighted	79 (59.8)	42 (51.2)	

## Data Availability

The data used to support the study are available from the corresponding author upon request.
